# Maternal environmental effects and climate‐smart seeds: unlocking epigenetic inheritance for crop innovation in the seed industry

**DOI:** 10.1111/tpj.70407

**Published:** 2025-08-08

**Authors:** Sophie Brunel‐Muguet, Miroslav Baránek, Sotirios Fragkostefanakis, Christopher Sauvage, Michal Lieberman‐Lazarovich, Stéphane Maury, Eirini Kaiserli, Na'ama Segal, Pilar S. Testillano, Jérôme Verdier

**Affiliations:** ^1^ Normandie Université, UNICAEN, INRAE, UMR 950 Ecophysiologie Végétale, Agronomie et nutritions N, C, S, Esplanade de la Paix CS14032 14032 Caen Cedex 5 France; ^2^ Mendeleum—Institute of Genetics, Faculty of Horticulture Mendel University in Brno Valtická 334 69144 Lednice Czech Republic; ^3^ Institute of Molecular Biosciences, Cell and Molecular Biology of Plants Goethe University Frankfurt Max‐von‐Laue Str9, 60u438 Frankfurt am Main Germany; ^4^ Syngenta SAS France 1228 Chemin de l'Hobit Saint Sauveur 31790 France; ^5^ Institute of Plant Sciences Agricultural Research Organization ‐ Volcani Institute 7505101 Rishon LeZion Israel; ^6^ Physiology, Ecology and Environment (P2e, ex LBLGC), INRAE University Orléans EA 1207 USC 1328, 45067 Orleans France; ^7^ School of Molecular Biosciences, College of Medical, Veterinary and Life Sciences University of Glasgow Glasgow G12 8QQ UK; ^8^ Israel Oceanographic & Limnological Research Ltd.—National Center for Mariculture Eilat 8811201 Israel; ^9^ Pollen Biotechnology of CropPlantsGroup Biological Research Center Margarita Salas (CIB) CSIC Madrid Spain; ^10^ Univ Angers Institut Agro, INRAE, IRHS, SFR QUASAV F‐49000 Angers France

**Keywords:** seeds, epigenetics, maternal, stress memory, germination, acclimation, intra/inter/transgenerational memory, seed priming

## Abstract

Seed production is facing a three‐fold challenge: ensuring food security, maintaining sustainability, and adapting to climate change. Although most efforts have focused on genetic breeding and crop management, additional levers need to be explored to optimize plant tolerance to the accelerating climate change. A groundbreaking approach will be to capitalize on the ability of plants to naturally adjust their responses to fluctuating environments during the crop cycle and transmit stress‐induced information to the next generation(s). This viewpoint aims at highlighting the potential application of maternal stress memory as a priming strategy to produce primed seedlots. This requires identifying the priming conditions among stress memory scenarios, defined according to the starting point of the new generation within the plant, that is, the fertilization. If the contribution of stress‐induced epigenetic‐associated mechanisms in inheritance patterns to promote germination and early growth development has been evidenced, the whole picture is not fully understood. Further investigations are required to characterize the maternally inherited plant stress imprints leading to higher stress tolerance of seedlots. Detailed characterization of the mechanisms of stress‐induced maternally heritable seed traits could provide novel targets for the seed industry and open new avenues to deploy the potential of maternal stress memory for enhancing seed performances.

## Significance Statement

Maternal stress memory is a reflection of the maternal environment to the offspring. Plant seeds can thus inherit tractable stress tolerance traits, meaning that the maternal environment allows the seeds to be primed. The mechanisms controlling the heritability of stress‐induced plant traits to the next generation offer opportunities to optimize seed production through the utilization of innovative epigenetically regulated breeding schemes.

## SEED PRODUCTION IN THE CONTEXT OF CLIMATE CHANGE

### Ongoing evolution of the seed market and compliance with quality standard for commercialization

Seeds are cornerstones for agriculture. Not only do they convey the genetic features for the crops to grow under a wide range of environmental conditions (seeds as agricultural inputs), but they also provide nutritional resources as food or feed (seeds as agricultural outputs). Over the last two decades, the seed market has tripled and is expected to surpass $100.13 billion by 2030 (Mordor Intelligence, n.d.) as a consequence of an increased demand to ensure food security and wider offers from the seed sector. In this context, the acceleration of climatic hazards has imposed an additional challenge to seed production as crops need to adapt to more intense, long lasting and/or more frequent environmental constraints without compromising the nutritional quality and germination ability of the seeds produced. Prior to their commercialization, seedlots go through a certification process following international standard tests on germination and purity. In addition to these criteria, seed vigor which is defined ‘the sum of those properties that determine the activity and performance of seedlots of acceptable germination in a wide range of environments’ (ISTA, 2021) is also a desired trait to assess as it reflects the susceptibility or tolerance of seedlots to environmental stresses under adverse and ever‐changing conditions (Bennett et al., 1992; Finch‐Savage & Bassel, 2016; Reed et al., 2022). Vigor tests can be requested by seed companies or performed in‐house to assess their stock and make marketing decisions, which is in line with the emergence of seed enhancement technologies (SETs) that aimed to promote seed vigor (Weissmann et al., 2023).

### The impact of climate change on seed performance

The increasing frequency of extreme climatic events over and across crop seasons is jeopardizing the agricultural sector and global food security. Yearly record‐breaking heat waves, droughts, and floods have been recorded worldwide (IPCC, 2021; WMO, 2022) and lead to seed yield penalties and quality losses of economically important seed crops (Lobell et al., 2015; Rötter et al., 2011; Schlenker & Roberts, 2009; Yawson & Adu, 2023). During the reproductive stages, heat waves and/or droughts impair bud flower development, anthesis, seed filling, and seed maturation, thereby not only affecting seed yield components but also seed composition and eventually seed germination performance and resilience to upcoming stresses (Fahad et al., 2017). Indeed, seed germination and seedling establishment are the first sensitive stages to a wide range of adverse conditions such as late frost in spring crops (Gu et al., 2008; Lamichhane, 2021; Zheng et al., 2012) or belated summer heat waves combined with low soil water reserve in winter crops (IPCC, 2021). Therefore, because seed germination ability is acquired during the reproductive stage, the conditions of production, that is, the environmental conditions on the mother plants, are determining (Bewley et al., 2013). It is now widely established that these conditions determine to some extent the offspring's phenotypic adaptation in a positive or negative way that reflects, respectively, the establishment of positive stress memory experienced by the maternal line or the amplification of penalizing effects on seed quality due to stressful events (Bezodis & Penfield, 2024; Crisp et al., 2016; Hilker et al., 2016; Hilker & Schmülling, 2019). A growing body of research has been exploring the impact of the environmental conditions on the mother plants on seed germination quality (Table 1) (see review Penfield & MacGregor, 2017). Seed dormancy and seed longevity are two components of seed germination quality, which are complex traits driven by environmental factors, mainly temperature over the reproductive stages. In general, high temperature exposure of maternal plants during seed maturation promotes seed germination vigor, while lower temperatures tend to increase the dormancy level, resulting in slower germination. Similarly, water stress during seed development leads to changes in seed dormancy (Fenner, 1991). Seed longevity was also shown to be influenced by suboptimal growth conditions, including heat, cold, and drought conditions during the late seed maturation phase in *Medicago truncatula* (Righetti et al., 2015) and many plant species (see review Leprince et al., 2016). To these non‐visible alterations of seed germination performance during seed maturation, higher seed moisture content at the end of seed maturity under wet conditions will lead to seed pre‐harvest sprouting (Gubler et al., 2005) or higher seed moisture in seed crops, which consequently causes a higher vulnerability to diseases in post‐harvest operations and yield loss (Maiorano et al., 2009; McCauley & Way, 2002).

**Table 1:** Overview of the effects of the maternal environment on conferring seed characteristics

Stress	Species	Stressing conditions over the plant cycle	Scenario^a^	Stress memory	Effects on seeds^b^	References
Heat	*Brassica napus* L.	G0 plants grown under long lasting high temperature (32.9/19.3) (day/night) from pod formation (GS81) until seed physiological maturity (GS99)	B	Intragenerational	Increased germination time courses at optimal (20°C) and suboptimal (5°C) temperatures Decreased seed storage capacity	Brunel‐Muguet et al. (2015)
Heat	*Brassica napus* L.	G0 plants under three modalities: 5‐day mild stress at the onset of pod filling (GS72),5‐day intense stress (GS81) or combination of both events	B	Intragenerational	Decreased seed dormancy (sucrose: oligosaccharides ratio)	Magno Massuia de Almeida *et al*. (2021)
Heat	*Brassica napus* L.	G0 and G1 plants under three modalities: 5‐day mild stress at the onset of pod filling (GS72), 5‐day intense stress (GS81) or combination of both events	C	Transgenerational	Increased germination time courses at optimal (20°C) and suboptimal (5°C) temperatures Decreased dormancy of seeds compared with seeds from non‐stressed plants over the two generations	Magno Massuia de Almeida *et al*. (2022)
Heat	*Triticum aestivum* L.	G0 plants subjected to 29°C/23°C (day/night) from anthesis to the beginning of grain filling	B	Intragenerational	Increased germination rate at low temperature (5°C) but not at optimal temperature (20°C)	Dürr et al. (2018)
Heat	*Pisum sativum* L.	G0 plants subjected to a range of day–night temperatures* from seed filling at last reproductive node until seeds reached 15% water content *28°C/23°C(day/night), 30°C/25°C or 35°C/30°C	B	Intragenerational	Lower germination rates at low temperature (5°C) but not at optimal temperature (20°C)	Dürr et al. (2018)
Heat and Drought	*Triticum durum* L.	G0 grown under water stress from the booting stage (soil water content at half of the field capacity) combined with heat stress at post‐anthesis (37/27 °C for 24 h at 5, 15, 25, 35, and 45 days)	A and B	Intergenerational for water stress Intragenerational for heat stress	Decrease in germination and early growth‐ related parameters under optimal temperature (26°C)	Liu et al. (2020)
Drought	*Brassica napus* L.	G0 plants subjected to 3‐week water withdrawal starting shortly before flowering	A and B	Inter‐ and intragenerational	Increased or decreased mean germination time (MGT) under non‐limiting conditions according to the genotype and unchanged or increased MGT under water stress	Hatzig et al. (2018)
Drought	*Helianthus annuus* L.	G0 plants subjected to water stress over seed development (full duration, before maturation until harvest, after maturation until harvest, end maturation until harvest)	B	Intragenerational	Decreased seed dormancy of seed after harvest Increased tolerance to low temperature (5°C), under water stress (−0.7 MPa) and hypoxia at germination	Vancostenoble et al. (2022)
Drought	*Glycine max* L.	G0 plants subjected to soil moisture stress during reproductive growth	C	Transgenerational	Reduced germination, seedling vigor and seed quality in the seed from G1	Wijewardana et al. (2019)
Drought	*Arachis hypogaea* L	G0 plants subjected to primed acclimation consisting of deficit irrigation during early vegetative growth followed by full supply	A	Intergenerational	High genotypic variability in germination and vigors characteristics	Racette et al. (2020)
Drought	*Oryza sativa* L.	Reduced water supply from tilling to seed filling over 10 generations	B	Transgenerational	Increased seed setting rate	Zheng et al. (2017)
Drought	*Piper umbellatum* L.	G0 grown either during rainy and dry seasons	A	Intragenerational	Greater germinability and longevity of rainy season seeds	Valentin‐Silva et al. (2023)
Drought	*Polygonum persicaria*	G0 and G1 plants subjected to a reduction of 50% soil capacity from seedling stage for 71 days (before self‐fertilization)	C	Transgenerational	Longer root system, deeper and faster root development in G2 seedlings from drought‐stressed grandparents (G0) or parents (G1)	Herman et al. (2012)

**Figure 1:**
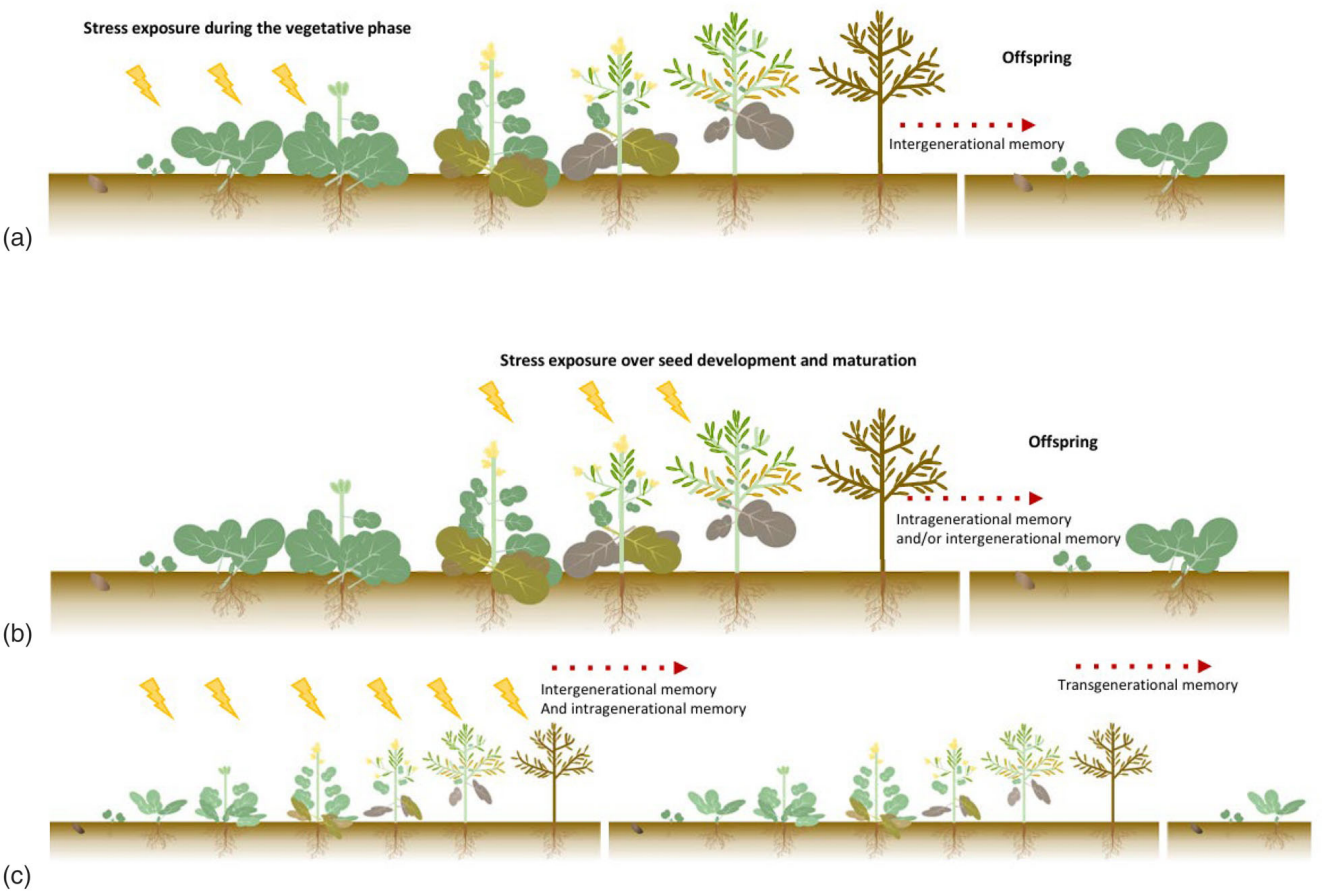
Stress scenarios according to the timing of the stress exposure(s). (a) Stress exposure affects the vegetative plant organs prior to fertilization. Intergenerational effects are mediated by (i) modifications of sink strengths due to a reduction in reproductive buds and pollen damage and (ii) epigenetic marks that are deposited in maternal tissue and eventually are inherited to the gametes by meiosis. (b) The first stress exposure affects reproductive organs after fertilization. Intergenerational memory effects are mediated by the effects of stress on both the vegetative plant organs (maternal origin) and developing seeds. When the source of the signal is the embryo tissue, these effects are intragenerational (somatic memory). Developing seeds are “prime” while still on the mother plant. (c) Transgenerational memory effects on seeds reflect the transmission of stress‐induced cues from the “grandparent” plants. Some authors distinguished intergenerational and transgenerational stress memory based on the number of stress‐free generations with detectable stress imprints (at least two for transgenerational, Lämke & Bäurle, 2017). In this viewpoint, we refer to transgenerational when the effect of the stress is observed on seeds/seedlings/plants from two generations (whether both were exposed to stress or only the first one).

Examples of the effects of environmental stresses (mainly high temperature and drought) during the mother and/or grandmother plants' cycle on seed germination quality and/or early standing. The provided examples aim to illustrate the different stress memory scenarios given in Figure 1.

^a^ Based on Figure 1.

^b^ In seeds/seedlings from stressed mother plants in comparison with seeds from unstressed plants OR in seeds/seedlings from stressed grandmother plants (and stressed or unstressed mother plants) when transgenerational.

### Conventional and emerging levers of acclimation based on the priming of seeds

Facing climate change has become central to seed companies and farms to ensure crop production and food security. Basically, adaptation to adverse conditions at crucial yield‐building stages relies on tight adjustments of crop management practices and on genetic improvement. Changes in crop management practices broaden, moving back or forward the sowing date and/or the use of early or late varieties, which overall aim to avoid coincidence between crop critical stages and expected climatic risks (Hampton et al., 2016) and even moving production sites, that is, crop switching (Rising & Devineni, 2020). Genetic improvement is another lever, for which genetic diversity is a prerequisite, but intense breeding programs following the Green Revolution largely contributed to its erosion, thus shrinking the accessibility of stress‐resilient germplasms in favor of input‐responsive varieties (Khoury et al., 2022). This observation has encouraged initiatives to investigate the resilience of traditional varieties (Longin & Würschum, 2016) and to backcross modern varieties with their wild ancestors, which are more resilient to adverse environmental conditions (a process known as rewilding) (Palmgren et al., 2015). More recently, the use of new genomic techniques (NGTs), such as genome editing, is an alternative way of breeding to develop climate change‐resilient crops (Karavolias et al., 2021; Pourkheirandish et al., 2020), even if the regulatory status of this topic is still actively discussed worldwide. In the context of seed production, another applied lever for the crops to acclimate to stressful conditions relies on the exploration of the priming of seed germination, called seed priming. Indeed, to attenuate the impacts of the environment on seed germination performances, post‐harvest treatments, called seed enhancement technologies (SETs), were developed by industry. One of the SETs is seed priming, which consists of a pre‐sowing treatment that involves soaking seeds in aqueous solutions to initiate the early stages of germination before planting (for review, Paparella et al., 2015). The fast completion of these early stages is definitely an asset for seed performance and seedling establishment. While the setting of the conditions for an optimized germination behavior is performed on post‐harvested seeds (i.e., seed priming), seeds can also be sensitized as a consequence of stress‐induced priming conditions during their development and filling on the mother plant. Hereafter, we differentiate seed priming (i.e., pre‐germination process) from stress priming (i.e., plant exposure to a mild stress that enhances its resilience to future severe stresses). Overall, both definitions describe conditions experienced by the seed at different developmental timings that must be explored to enhance seed and plant performance with respect to stress tolerance. Both seed priming and stress priming involve a memory effect via epigenetic, hormonal, and metabolic adjustments. In this manuscript, we discuss the potential of stress priming through inherited information from the mother plants for improving seed early performances.

## TO WHAT EXTENT DOES THE CLIMATIC HISTORY OF THE MOTHER PLANT IMPACT THE EARLY GROWTH STAGES OF THE OFFSPRING?

### The role of the maternal growth environment on seed lot germination performance

The maternal environmental effects have been defined as ‘the influence of the maternal parent's external ecological environment on the phenotype of its progeny’ (Donohue, 2009, Box 1). These effects have been categorized into maternal genetic effects resulting from plastids inheritance strictly, effects of the triploid endosperm (two‐thirds of the genotype being of maternal origin), effects of maternal tissues such as seed coat and seed reserve compounds and eventually the effects of the mother plant on its ability and influence on the seed dispersal. Maternal provisioning, which involves the effects of maternal tissues, is key, since it allows the embryo to be nurtured until the early seedling stage, providing the necessary hormones, proteins and transcripts for its development.

Box 1 **Key definitions**

Epigenetic inheritance refers to the inheritance of a phenotype in a manner that does not alter the DNA sequence and that remains self‐perpetuating in the absence of the initial stimulus that caused the phenotype in the parental cell or organism. The inheritance is somatic when it refers to mitotically inherited epigenetic marks and inter‐ or transgenerational when it refers to meiotically inherited epigenetic marks.

Epigenetic stress memory refers to the processes that allow stress‐induced epigenetic marks to be induced and erased upon later stress exposure.

Heritable epigenetic modifications describe changes that occur in biological molecules and/or their arrangements that are transmitted across generations without altering the genome sequence. Past definitions have been similarly broader or narrower, with a focus on particular chemical modifications (e.g., DNA methylation) or changes in regulators (e.g., small RNAs). The concepts outlined here apply to both types of definitions.

Maternal effects define the causal effects of the maternal genotype or phenotype on the offspring phenotype.

Maternal environmental effects specify the influence of external cues to which the maternal parents are exposed, on the offspring behavior and/or phenotype. Maternal environmental effects are known to impact evolutionary processes such as phenotypic plasticity in responses to environmental recurring constraints.

Maternal stress memory effects refer to the inherited information that is triggered by environmental signals perceived by the mother plant. Following this definition, inter‐ or intragenerational (or somatic) maternal stress memory refers to observed effects on the next generation when the stress signal occurs before or after the fertilization of the female gametophyte, respectively.

Priming is the phenomenon that reflects sensitization following exposure to a stimulus. The so‐called priming stress induces information that allows the plant and/or the offspring to be “prime,” that is, to respond in a more rapid and effective way to a later stimulus or triggering stress. The priming stress and the triggering stress can be of the same or of different nature.

Epibreeding refers to the process of plant breeding that exploits epigenetic variations to enhance desirable traits. Unlike traditional breeding methods that rely solely on genetic variations (changes in DNA sequence), epibreeding focuses on heritable changes in gene expression that do not involve alterations to the underlying DNA sequence. These changes can be induced or selected to improve traits such as stress tolerance, yield, and disease resistance.

As illustrated in Table 1, the maternal environment, reflecting exposure to stressful conditions, highly influences seed quality traits and seedling growth of offspring. Evidence for its impact in an agronomic context was observed in intercropping field assays (Chen et al., 2020), where shaded soybean plants produced seedlots that germinated faster in the dark. This was explained by increased expression of genes involved in gibberellic acid (GA) biosynthesis and decreased expression of abscisic acid (ABA) biosynthesis genes, highlighting an adaptive environmentally induced parental effect. Recent studies have reported that prolonged heat waves (Brunel‐Muguet et al., 2015) or short heat events during pod formation affect seed germination at sub‐ and optimal temperatures even after two heat‐stressed plant generations (Magno Massuia de Almeida *et al*., 2022), leading to increased germination rates. In crops like wheat and pea, higher germination rates were also observed under low temperatures for seeds from heat‐stressed plants during seed filling (Dürr et al., 2018). Studies also revealed positive effects of short drought episodes on offspring resilience (Table 1). For instance, in barley (Nosalewicz et al., 2016), peanut (Rowland et al., 2012), rice (Zheng et al., 2017) or *Secale sylvestre* (Mojzes et al., 2021), early growth traits of offspring from drought‐stressed mother plants were enhanced, supporting robust seedling establishment. *Polygonum persicaria* exposed to two drought‐stressed generations also showed improved seedling growth (Herman et al., 2012). In sunflower, drought exposure of maternal lines positively affected seed germination not only under water deficit, but also under low temperature and hypoxia stresses (Vancostenoble et al., 2022). These findings suggest cross‐tolerance or cross‐hardening effects, as reviewed in other studies (Hossain et al., 2018; Mittler, 2006), unveiling the potential of primed acclimation to multiple stresses. However, this requires investigation in species‐ and stress‐dependent manners. Indeed, studies highlighted contrasting inherited priming responses in peanut (Racette et al., 2020) and Arabidopsis (Suter & Widmer, 2013). Moreover, combined stresses like drought and heat may yield opposite outcomes for the next generation's resilience compared with individual stress (Sehgal et al., 2018). For example, in wheat, heat and drought after booting negatively impacted germination and seed vigor, even under optimal temperature (Liu et al., 2020), whereas in *Petunia*, environmental stresses including water limitation, high salinity, and high temperature positively affected seed germination and seedling vigor (Nguyen et al., 2021).

Overall, although not exhaustive, these studies indicate that germination capacity and seedling growth of seeds from stressed mother plants were positively or negatively impacted in many species, without presuming the underlying processes and levels of regulation. The effect is genotype‐dependent, with the magnitude and nature of the impact tied to the timing, intensity, and duration of stress experienced by maternal plants or previous generations. The maternal plant's growth conditions reflect how stress memory‐related processes are sustained through stress‐triggered modifications on post‐transcriptional processes that arise in the mother plants or in the seeds already present. As specified earlier, these environmental maternal effects are of different origins and can be mediated by epigenetic imprinting, biochemical variation, and/or hormone level changes (Bonduriansky & Day, 2009; Liu et al., 2022).

In the next section, we focus on stress‐induced epigenetic regulations in mother plants to harness field conditions for producing stress‐resilient seed lots.

### The contribution of stress‐induced epigenetic regulations to maternal effects

#### Stress‐induced epigenetic mechanisms

Epigenetic mechanisms are key components of stress responses to fluctuating environments, allowing the plants to timely adjust their phenology and metabolic responses (Kakoulidou et al., 2021). These chemical modifications on the DNA bases and histone proteins are often reversible and can be inherited through generations; therefore, they contribute to short‐ to medium‐term epigenetic memory effects. DNA methylation that mainly occurs in symmetric CG and CHG contexts or asymmetric CHH context represses gene expression by preventing the binding of transcription factors to the DNA or by affecting the recruitment of chromatin remodeling proteins that alter 3D chromatin structure (Gao et al., 2014). The dynamics of DNA (de)methylation are regulated by the developmental/environmental cues and under the control of writer/eraser guided by non‐coding RNAs (Kumar & Mohapatra, 2021; Zhang et al., 2018). Over seed development, CG methylation is globally stable compared with CHG and CHH methylation (Hsieh et al., 2016), which allows greater stability regarding meiotic resetting of methylation (Mathieu et al., 2007; Paszkowski & Grossniklaus, 2011). Histone post‐translational modifications (PTMs) are another epigenetic‐associated mechanism impacting chromatin 3D structure and genome DNA accessibility. Many PTMs (e.g. acetylation, ubiquitination, phosphorylation and methylation) on histones 3, 4, 2A, and 2B and histone variants control gene transcription, representing either active or repressive histone marks. These PTMs are set by the action of various protein modifying enzymes (see review Ueda & Seki, 2020), which allow dynamic and reversible changes, enabling cells to respond quickly to environmental signals (for reviews, Zhao et al., 2019; Wu et al., 2023). As examples, the most frequently described histone PTMs involved in seed development are H3K27me3, H3K4me3, or H3K9me2 (see review Haider & Farrona, 2024). Related to seed vigor, it has been well documented the contribution of histone PTMs induced by maternal conditions on dormancy release. As an example, Molitor et al. (2014) demonstrated that the DOG1 (DELAY OF GERMINATION1) gene expression was regulated by changes in H3K4me3 and H3K27me3 histone marks, such that over dormancy relief, the repressive mark H3K27me3 was gradually accumulated. These epigenetic marks on DOG1 act as a thermal monitoring mechanism that triggers light repression of dormancy (Footitt et al., 2015).

Histone PTMs and DNA methylation are intertwined mechanisms that regulate gene expression. For example, DNA methylation can recruit proteins that modify histones, leading to a more compact chromatin structure. Another key player in the epigenetic‐based regulatory response is non‐coding RNAs such as small interfering RNAs (siRNAs) that regulate gene expression through gene silencing or chromatin remodeling (Ramirez‐Prado et al., 2017). Small RNAs control the RNA‐directed DNA methylation (RdDM) pathway, which is usually associated with the transcriptional regulation of stress‐responsive genes. As a consequence, they can mediate DNA methylation patterns, which can be meiosis‐inherited and eventually contribute to epigenetic effects on gene expression and phenotype of the offspring (Erdmann & Picard, 2020). Small RNAs are phloem‐mobile and can potentially be transferred from vegetative to reproductive organs and eventually developing seeds to further induce stable epigenetic changes in the progeny genome (Zhan and Meyers, 2023).

#### Inheritance of epigenetic marks

Epigenetic inheritance refers to epigenetic modifications maintained within the individual but reset between generations or inherited across generations, making them dynamic on multiple timescales (Hemenway & Gerhing, 2023). Inheritance over the plant life, that is, intragenerational memory, relies on the maintenance of epigenetic marks through mitoses. During mitosis, the epigenetic landscape is largely preserved with minor changes. Regarding histone modifications, half of the histones deposited after mitosis derive from the parental chromatin, and the other half is newly synthesized to maintain the epigenetic status (Budhavarapu et al., 2013). As reviewed by Wang and Higgins (2013), while most histone modifications are maintained (e.g., H3K4me2/3, H3K9me2/3, H3K27me3), some decrease (e.g., H2AK119ub, H3K9ac) during mitosis in plants. DNA methylation marks are also inherited during replication in all contexts. However, in clonally propagated maize and rice, Stroud et al. (2013) and Han et al. (2018) showed stable methylation inheritance through regenerants alongside global methylation loss (mainly CHH context), likely due to less efficient RdDM pathway in fast cell cycles (Borges et al., 2021). Ibañez and Quadrana (2023) also suggested that, in clonally propagated plants, the lack of fertilization followed by embryogenesis, where DNA methylation reinforcement occurs, generally leads to progressive, irreversible CHH loss. Stress‐induced epigenetic regulations are usually reset during plant gametogenesis and after fertilization as memory mechanisms come with unnecessary metabolic costs if the subsequent generations face no similar stresses (Hemenway & Gehring, 2023). Memory from vegetative plant organs from parents is typically erased during embryogenesis due to epigenome reprogramming (Ingouff et al., 2010). For instance, the flowering repressor *FLC*, silenced by vernalization, is reprogrammed during embryogenesis to prevent transgenerational inheritance and allow the environmental control of flowering (Choi et al., 2009; Sheldon et al., 2008; Tao et al., 2017). However, environmentally induced epigenetic marks can be passed on, that is, inter/transgenerational memory, through complex regulations during the gamete to zygote transition (Bilichak & Kovalchuk, 2016; Jo & Nodine, 2024). The inter/transgenerational inheritance of the epigenetic landscape during meiosis and fertilization involves maintenance, resetting, and selective transmission of DNA methylation and histone PTMs. Insights into these processes have been compiled by Gehring (2019), Ono and Kinoshita (2021), Tirot and Jullien (2022), Ibañez and Quadrana (2023), and Jo and Nodine (2024). These studies underscore the complexity of inheritance in seed plants as seeds are a complex structure that contain different genetic origins tissues, that is, the seed coat, which is purely maternal tissue; the embryo, which is the next generation; and the endosperm, which is a product of double fertilization. All three of these tissues could affect seed germination/seedling establishment via maternal effects. However, only the embryo can transmit intergenerational/transgenerational effects that persist significantly beyond the seedling stage. These studies reveal dynamic epigenome changes during reproductive development, with memory effects depending on gamete origin and mark type. Plants show no global erasure of DNA methylation during reproduction. Instead, epigenetic marks are passively lost or inherited through the female gamete, while male gametes contribute less due to extensive epigenetic reprogramming, largely driven by DNA glycosylases such as DEMETER (Batista & Köhler, 2020; Calarco et al., 2012). Regarding stress memory, heritability of DNA methylation changes is complex. Wibowo et al. (2016) showed osmotic stress‐induced changes in Arabidopsis were inherited through the maternal lineage, whereas Van Dooren et al. (2020) found no inheritance under salt stress.

Though rare, meiosis‐inherited stress priming has been shown in several contexts (Lagiotis et al., 2023). In rice, multigenerational drought exposures altered methylation in guard cells, affecting photosynthesis and gas exchange, aiding offspring adaptation (Zheng et al., 2017). Similarly, multigenerational heat stress enhanced phenotypic acclimation and DNA methylation frequency (Yadav et al., 2022). In Arabidopsis, drought‐exposed epigenetic recombinant inbred lines (*epiRILs*) supported a causal link between methylation and heritable traits (Zhang et al., 2013). Hypomethylated loci (epiQTLs) were also found to quantitatively control mildew resistance (Furci et al., 2019). Some naturally occurring epialleles were also identified. For instance, in tomato, the colorless non‐ripening (cnr) mutant exhibits a non‐ripening fruit phenotype. This mutation is caused by a stable epiallele characterized by the hypermethylation of the promoter of the SQUAMOSA promoter binding protein–like gene (SBP‐box gene) (Manning et al., 2006). In Arabidopsis, a naturally occurring retrotransposon named “NMR19” (naturally occurring DNA methylation variation region 19) leads to variation in leaf senescence. This retrotransposon has a stably inherited DNA methylation status that influences the expression of the PHEOPHYTIN PHEOPHORBIDE HYDROLASE (PPH) gene (He et al., 2018). In Vatov and Gechev (2025), examples in rice and tobacco showed how stress‐induced cytosine methylation and resistance traits are regulated by transposable elements (TEs). In biotic contexts, small RNAs were shown to mediate inherited defense. For instance, Rasmann et al. (2012) documented transgenerational herbivory resistance relying on phloem‐mobile small RNAs. Such small RNAs also influenced germination and seedling vigor under water deficit and heat stress in wheat (Liu et al., 2020) and in perennials like *Pinus radiata* Lamelas et al., 2024). These examples demonstrate that even though epiRILs are experimental constructs, the underlying principles of heritable epigenetic variation (epialleles) also occur naturally. They also highlight how DNA methylation variations, induced by environmental (abiotic) stresses, significantly contribute to phenotypic diversity and adaptation in plant populations.

Although epigenetic marks tied to transgenerational inheritance are scarce and underlying mechanisms remain elusive, mitotically inherited marks may be sufficient in contexts where the stress occurs during the reproductive phase, as detailed below.

### Stress‐specific priming scenarios impacting offspring performance

As illustrated in Table 1, seed characteristics are tightly shaped by environmental conditions. However, the precise nature of these modifications affecting seed performance remains largely unknown and depends on the timing of stress exposure in mother plants. Depending on when stress occurs during the plant cycle, these modifications are either direct (i.e., when seeds develop during stress), indirect (i.e., when stress occurs prior to the reproductive phase) or a combination of both when spanning the vegetative and reproductive phases (Figure 1). This distinction is important when describing intra‐, inter‐, or transgenerational stress memory effects, especially with a focus on epigenetic inheritance (Box 1, Table 1). The following description of memory types clarifies a framework linking stress timing to phenological stages and corresponding epigenetic regulation. Intragenerational stress memory (somatic memory) refers to epigenetic information triggered by environmental signals, transmitted cell to cell through DNA methylation and histone PTMs, enabling adaptation within‐generation acclimation. Transmission of this epigenetic information to daughter cells occurs during mitosis.

Additionally, plants exposed to environmental stress can produce offspring with memory effects and increased stress resilience—termed intergenerational or parental memory (Kambona et al., 2023). The concept of intergenerational memory is relatively recent (Lämke & Bäurle, 2017). Such claims require careful analysis, particularly when stress is applied to plants with developing seeds. It is essential to determine whether stress affects somatic tissues, germline tissues, or seeds (Figure 1a). The former does not reflect true intergenerational effects but instead direct exposure of the mother plant carrying embryos, relating to somatic memory. When stress occurs in plants with developing seeds, distinguishing between intra‐ and intergenerational memory becomes difficult (Figure 1b) However, although it is difficult to identify the origin of the offspring memory when it is established during flowering/seed production, crossing experiments between stressed female parent plant and unstressed male parent plant (irrespectively) can help overcome this issue and identify the parent‐of‐origin of the priming information. In Wibowo et al. (2016), reciprocal crosses with non‐primed plants can differentiate between information from maternal and paternal origin. Finally, transgenerational effects represent the third memory type, requiring specific conditions for transmission to further generations (Figure 1c), also called “grandparental” memory (Kambona et al., 2023). In both inter‐ and transgenerational memory, stress‐initiated epigenetic mechanisms must be transmitted through meiosis and fertilization.

## GAPS AND CHALLENGES ON THE USE OF STRESS PRIMING TO ENHANCE SEED PERFORMANCE

### Need to predict how the maternal environment affects offspring performance

In this report, we explored the impacts of the maternal environment on seeds and early growth traits, and discussed the relevance of the stress history of the mother plant that may influence the biological and epigenetic features of the offspring. Key points to avoid misinterpretation of the analysis of epigenetic stress memory effects are highlighted. First, fertilization must be considered as the starting point of the next generation. This would help analyze the stress‐memory effects on the offspring according to the organ that was exposed to stress on the mother plant (Figure 1). A consensual definition of maternal stress memory effects on the offspring would require a distinction of the timing of stress occurrence, for example, before fertilization, thus being intergenerational maternal stress memory effects (Figure 1a), or after fertilization, being intragenerational maternal stress memory effects combined with potential intergenerational effects (Figure 1b). However, in addition to this time mark, the source from where the priming signals have arisen is also determining since post‐fertilization memory could still be intergenerational if the stress‐induced signals (e.g., sRNAs) are transferred from maternal to embryo tissues. Second, a clear definition of transgenerational stress memory needs to be stated because most often intergenerational and transgenerational are used irrespectively. Indeed, depending on studies, it refers to situations in which either stressing cues have an impact on progenies or stressing cues are extended to at least two stress‐free generations (Lämke & Bäurle, 2017; Figure 1c). We argue that this last definition should be used to clarify the number of stress‐exposed generations and so the “recurrence” of the stress over time.

### Challenges to harness stress memory to improve seed performance

**Figure 2:**
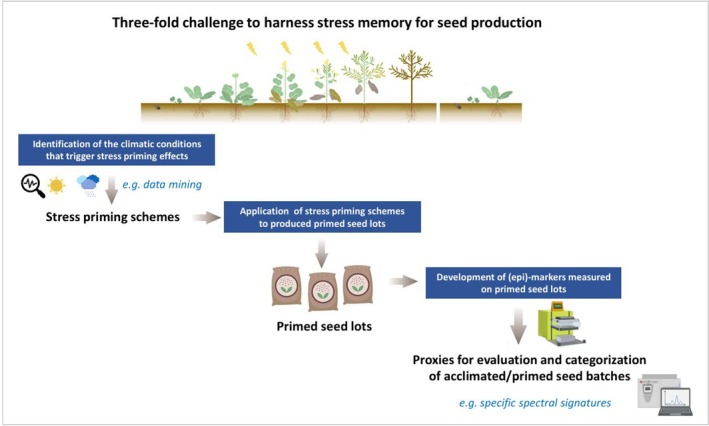
A roadmap for exploring stress priming for seed performance in seed production. Illustrative items (seed lot bags, climate conditions, lab equipments) ©Biorender.

In contrast to the conventional levers to produce stress‐resilient seedlots or to avoid stressful conditions during germination and seedling establishment, an overlooked lever is environment‐induced acclimatization to stressful conditions, which relies on the inherited features from the stressed mother plant. Therefore, opportunities to harness stress priming emerge as a groundbreaking option to produce more resilient seed batches or climate‐smart seedlots (i.e., more adapted to a specific climate). To achieve this goal, three main challenges need to be overcome: (i) the thorough identification of the climatic conditions that trigger stress priming effects (i.e., stress priming schemes), (ii) the application of the stress priming scheme to produce primed seedlots, and (iii) the development of markers or specific proxies that reflect inherited stress priming to evaluate and categorize primed seedlots (Figure 2). Finally, another challenge is that the reliability and reproducibility across species and environments of maternal stress memory are not yet clearly established and need to be validated.

#### Deciphering conditions of mother plants stress priming for seed production

The underlying idea of deciphering climatic conditions of seed production on mother plants is to correlate specific environmental scenarios with seed performance. Indeed, as previously illustrated in Table 1, there is not a single acclimation or sensitization scheme usable for seed performance. Priming effects (intra, inter or transgenerational) depend on the stress sequence's features on parent or grandparent plants —that is, intensity, duration, frequency, and timing of stress exposure(s)‐ and possible interactions between co‐occurring stresses that eventually lead to short‐term inheritance (over the crop cycle) or inter‐ and transgenerational inheritance, epigenetically driven. These characteristics are not only key for inducing stress priming but also for its maintenance throughout the plant cycle and transmission to the next generations. For instance, Magno Massuia de Almeida et al. (2021) demonstrated from greenhouse experiments in oilseed rape that a 5‐day recovery between mild and intense heat stress led to a negative additive effect. By contrast, a gradual increase in temperature before intense stress, without recovery, showed a priming effect, mitigating the intense heat stress (Delamare et al., 2025). These observations raise the question of suitable stress memory duration and intensity to expect priming without negative drawbacks. Although few studies have focused on these aspects, Lämke and Bäurle (2017) summarized the maximal duration of epigenetic memory occurring in somatic and transgenerational stress memory, highlighting variability according to the nature of stress (e.g. drought, heat, osmotic stress), its characteristics (duration, intensity, frequency), which determine deposition and maintenance of specific epigenetic marks. In this context of sequential or combined multistress, the maternal environmental effects are not straightforward; instead, they result from a combination of various climatic events characteristics and their interactions. Therefore, the emerging area of artificial intelligence, including data mining approaches, offers perspectives to analyze climatic sequences, identify key features that induce stress‐priming effects on the progeny, and eventually produce predictive models of seed performance according to the climatic profile over the mother plant's life cycle (Magno Massuia de Almeida et al., 2020, Figure 2). Genotype‐dependent effects add a layer of complexity that also justifies mining extensive datasets.

#### Pre‐ and post‐harvest application of the stress priming schemes to prime seeds

For farmers and breeding companies, the identification of stress priming schemes is an important step, but the proper application of them to induce such effects could still be challenging. Indeed, primed acclimation in a crop management context usually consists of specific short, mild stress treatment(s) that induce transient molecular and metabolic alterations or long‐term changes in chromatin structure that enable a more rigorous response to more severe stresses (Liu et al., 2022; Vincent et al., 2015). However, because seed production is mostly achieved in the field, applying suitable priming conditions *in situ* is very limited. In the case of water limitation, the use of deficit irrigation timed to crop developmental periods was shown to prime the plants to later more intense water deficits (Rowland et al., 2012, Vincent et al., 2020). Therefore, realistic water management strategies in the field can be designed to explore priming effects. Under strictly uncontrolled environmental factors (e.g., low, high temperatures, water excess, shading), another approach relies on the identification of primed seedlots, that is, seedlots with improved seed germination and vigor characteristics inherited from the maternal environment. This not only requires having knowledge on the features that make a priming sequence but also proxies that measure these beneficial maternal environmental effects on the seeds. One possibility would rely on post‐harvest application of stress priming schemes in fully controlled conditions to explore their efficiencies on mature seedlots (Figure 2).

#### Development of (epi)markers to screen and categorize more resilient seed batches

As previously stated, the potential exploitation of epigenetically driven maternal stress memory could be bioengineered by the seed industry to generate better performing seedlots in response to a specific environment and stress exposure during seed production. Therefore, the next challenge is to predict if a seedlot is primed or not to a specific stress (Figure 2). Overcoming this challenge relies on *a posteriori* analysis of the growth conditions on the mother plants, aiming at highlighting the stress climatic features that induce the inheritance of positive maternal stress memory on the offspring. Through this approach, epigenetic marks (epimarkers) such as hypo‐ or hyper‐methylated DNA regions need to be identified under specific climatic conditions (*i.e*. identified as sensitization schemes). The identification of such seed epigenetic signatures will represent a useful tool to predict more resilient or climate‐smart seedlots, more adapted to specific climates to anticipate sowing stressing conditions. Not only will these (epi)markers be used for the identification of tolerant/resistant/resilient seedlots with higher performance, but also for breeding programs. Indeed, epigenetic variants represent an additional and timely resource for crop breeding, known as “epibreeding” (Box 1) to sustain genetic gain. It is essential to understand that epigenetic marks that are necessarily meiotically inherited are not a prerequisite to this approach, as somatic memory acquired on seed‐bearing mother plants would provide the required epimarkers to identify tolerant/resistant/resilient seedlots.

## A ROADMAP TO DEPLOY THE POTENTIAL OF MATERNAL STRESS MEMORY FOR ENHANCING SEED PERFORMANCE

As novel technologies stemming from academic research, the transfer of knowledge on plant stress memory faces hurdles before resonating with research and development divisions in the seed industry. Taking advantage of epigenetics in crop breeding will be challenging; so far, few studies have demonstrated successful applications (see review Tonosaki et al., 2022). By contrast, technologies such as genomic selection were rapidly adopted from livestock to plant breeding given the high genetic gains they offered. Similarly, translating epigenetic potential into measurable gains may be key for implementing maternal stress memory in seed production and breeding schemes, though scientific and technical obstacles remain. In summary, several challenges for academic and private sectors outline a roadmap for deploying epigenetic‐driven maternal stress memory in the seed sector:

1. Significant effort is needed to identify climatic profiles that induce positive maternal memory effects on seed performance. Large agroclimatic datasets must be explored to pinpoint conditions producing tolerant seedlots, using predictive modeling approaches such as data mining (Figure 2, Magno Massuia de Almeida et al., 2020). This approach— that ignores the potential epigenetic cause— must also account for multistress conditions that reflect realistic field environments, where stresses act synergistically, additively, or antagonistically.
2. More scientific knowledge is needed to identify efficient epigenetic markers (mainly DNA methylation) for breeding and/or plant acclimation through priming. Additionally, the predictability and heritability of these epigenetic markers are discussed, as stress memory does not always have an epigenetic basis (Liu et al., 2022). In Arabidopsis plants, an increase in DNA methylation levels was shown to be induced upon metabolic stress, but this effect was suppressed in the developing seeds, hence blocking memory processes (Girija et al., 2023). This scenario highlights the complexity of stress memory mechanisms on multiple timescales.
3. Technical development is essential to detect, track, and control the stability and heritability of epigenetic markers using high‐throughput, reliable, and low‐cost genotyping assays as competitive as traditional genetic markers. One option is using “cheaper” proxies such as changes in seed metabolite contents, indirectly reflecting maternal stress memory. This could be monitored by advanced technologies like near‐infrared spectroscopy coupled with deep learning (Vasseur et al., 2022) to track memory effects (Figure 2).
4. Clear regulations are needed to define how to commercialize primed/acclimated crops.

## CONCLUSION

Several opportunities for the private seed industry emerge from the aforementioned stress maternal effects on seeds and the increasing knowledge acquired on its epigenetic drivers. Evidence for mother plant‐inherited information passing to progenies emerges as a genuine tool for crop acclimation to recurring stresses within and over the seasons. This so‐called maternal stress memory highlights the possibility to produce acclimated/primed seeds and crops through epigenetic regulations. These features provide guidance and opportunities for plant breeders and seed companies to potentially harness pre‐ and post‐harvest sensitization schemes and to identify and use epigenetic markers, such as DNA methylation at specific loci of primed seedlots. In this viewpoint, we have made several recommendations that highlight where steps can be taken for the seed industry adoption and the public acceptance of such improved seedlots and varieties. They mainly deal with a deeper understanding of maternal stress memory drivers along with how to control and implement such drivers in seed production. We urge geneticists, agronomists, breeders, and modelers to seize this opportunity to speed up research for acclimated crops to the ongoing climatic challenges.

## Author Contributions

SBM and JV conceived the ideas, initiated the study, and wrote the sections. SBM made the Figures, and EK edited them. MB, SF, and CS provided significant written input on the maternal stress memory and epigenetics sections. SM added specific comments on the boxes' contents and the general organization of titles. EK edited the whole manuscript. All authors contributed critically to the drafts and gave final approval for publication.

## Data Availability

Data sharing is not applicable to this article as no new data were created or analyzed in this study.
